# MultiP-Apo: A Multilabel Predictor for Identifying Subcellular Locations of Apoptosis Proteins

**DOI:** 10.1155/2017/9183796

**Published:** 2017-07-04

**Authors:** Xiao Wang, Hui Li, Rong Wang, Qiuwen Zhang, Weiwei Zhang, Yong Gan

**Affiliations:** School of Computer and Communication Engineering, Zhengzhou University of Light Industry, Zhengzhou 450002, China

## Abstract

Apoptosis proteins play an important role in the mechanism of programmed cell death. Predicting subcellular localization of apoptosis proteins is an essential step to understand their functions and identify drugs target. Many computational prediction methods have been developed for apoptosis protein subcellular localization. However, these existing works only focus on the proteins that have one location; proteins with multiple locations are either not considered or assumed as not existing when constructing prediction models, so that they cannot completely predict all the locations of the apoptosis proteins with multiple locations. To address this problem, this paper proposes a novel multilabel predictor named MultiP-Apo, which can predict not only apoptosis proteins with single subcellular location but also those with multiple subcellular locations. Specifically, given a query protein, GO-based feature extraction method is used to extract its feature vector. Subsequently, the GO feature vector is classified by a new multilabel classifier based on the label-specific features. It is the first multilabel predictor ever established for identifying subcellular locations of multilocation apoptosis proteins. As an initial study, MultiP-Apo achieves an overall accuracy of 58.49% by jackknife test, which indicates that our proposed predictor may become a very useful high-throughput tool in this area.

## 1. Introduction

Apoptosis or programmed cell death is an autonomic ordered death process under certain physiological and pathological conditions in organisms. It maintains normal tissue homeostasis by keeping a balance between cell proliferation and death. When the regulation of cell apoptosis is disordered, diseases such as tumor, autoimmune diseases, and neurodegenerative diseases will emerge [1–3]. Apoptosis proteins play critical roles in the mechanism of programmed cell death. Identification of subcellular locations of apoptosis proteins could help us understand apoptosis mechanism [4]. During the last decade, there have existed many excellent prediction methods based on machine learning for apoptosis protein subcellular localization. In general, these works have three major steps: (1) construct or select a benchmark dataset for training and testing the predictor, (2) extract the important biological characteristics contained in the protein samples, (3) and introduce or develop a new machine learning algorithm.

In the first step, three benchmark datasets, ZD98 [4], ZW225 [5], and CL317 [6, 7], are the most widely used for apoptosis protein subcellular localization prediction. The ZD98 dataset has 98 apoptosis proteins and four subcellular locations, which consists of 43 cytoplasmic proteins, 30 plasma membrane-bound proteins, 13 mitochondrial proteins, and 12 other proteins. The ZW225 dataset contains four subcellular locations and 225 apoptosis proteins; they are 41 nuclear proteins, 70 cytoplasmic proteins, 25 mitochondrial proteins, and 89 membrane proteins. The CL317 dataset is the latest and largest existing dataset, which includes 112 cytoplasmic proteins, 55 membrane proteins, 34 mitochondrial proteins, 17 secreted proteins, 52 nuclear proteins, and 47 endoplasmic reticulum proteins. In the second step, many methods have been used to extract core and essential features of the apoptosis protein samples, such as amino acid composition [8], pseudo-amino-acid composition [6, 7, 9–12], group weight coding [5], distance frequency [13], autocovariance transformation based on position-specific score matrix (PSSM-AC) [14], and Gene Ontology (GO) annotation information [15]. In the last step, some common machine learning algorithms, for example, support vector machine (SVM) [13, 14, 16], fuzzy k-nearest neighbor (FKNN) [9, 10], and ensemble learning [17, 18], have been used to perform the prediction.

However, there are the following drawbacks in aforementioned works. (1) These prediction models are only applicable for the proteins that have one subcellular location. For apoptosis proteins with multiple locations, so far no models can completely and correctly predict all their subcellular locations. (2) Apoptosis proteins in the three benchmark datasets only have one subcellular location, but, to our best knowledge, they may be annotated as more than one location in the UniProtKB database; there is no dataset containing apoptosis proteins with multiple locations for subcellular localization prediction by now. (3) Predicting subcellular locations for apoptosis proteins with multiple locations is a multilabel learning problem; machine learning classifiers mentioned above cannot be directly applied to dealing with a multilabel problem. Proteins with multiple locations should be highly concerned, because they may have some very special biological functions worthy of in-depth research. Unfortunately, previous researches in the field are limited to the prediction of proteins with only single location. They generally constructed prediction models based on the assumption that multilocation proteins do not exist.

To address this problem, a new multilabel predictor, named MultiP-Apo, is proposed, which can predict not only apoptosis proteins with single subcellular location but also those with multiple subcellular locations. Firstly, a new benchmark dataset, MSapo518, is constructed, which contains 518 apoptosis proteins with both single and multiple subcellular locations. To expand the prediction range, the new dataset increased two new subcellular locations on the basis of subcellular location included in the CL317 dataset. Secondly, GO annotation information of the homologous proteins of apoptosis proteins is used to represent proteins, and a GO subspace is constructed by selecting a set of relevant GO terms from all the GO terms in GO database to avoid the curse of dimensionality. Thirdly, a new multilabel algorithm is presented as the prediction engine by utilizing the label-specific features. Finally, an online web server for MultiP-Apo is developed, which is freely accessible at http://biomed.zzuli.edu.cn/bioinfo/multip-apo/.

## 2. Materials and Methods

### 2.1. Dataset

In order to establish a high quality benchmark dataset, all apoptosis protein sequences used in the current study were collected from the UniProtKB/Swiss-Prot database (released on 04 July 2016) according to the following steps:

1. “Apoptosis” was used as the keyword to search the Swiss-Prot database; only the apoptosis protein sequences were collected.
2. Those protein sequences annotated with “fragment” were excluded, and the sequences with less than 50 amino acid residues were also excluded because they might belong to fragments.
3. Those proteins whose subcellular locations were annotated by experiment were collected, and the subcellular locations of proteins annotated with “by similarity” were excluded, because these subcellular locations were inferred from the homologous proteins.
4. The protein sequences including ambiguous or uncertain letters, like “B,” “X,” or “Z,” were excluded.

**Figure 1:**
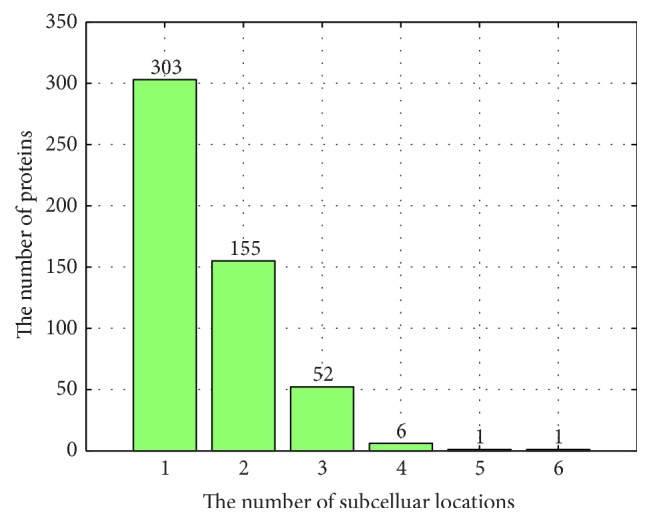
The distribution of proteins with different number of subcellular locations.

After the above four processes, we obtained the benchmark dataset MSapo518 including 518 different apoptosis proteins covered in the following 8 main subcellular locations, cytoplasm, membrane, secreted, mitochondrion, nucleus, endosome, endoplasmic reticulum, and Golgi apparatus. Although homology bias of the dataset might have an effect on the performance of the predictor, we still decided not to further reduce the number of proteins in the dataset because the quantity of apoptosis proteins annotated by manual experiment was very few. The number of apoptosis proteins belonging to each subcellular location is given in Table 1. Among these proteins, 303 proteins occur in one subcellular location, 155 in two locations, 52 in three locations, 6 in four locations, 1 in five locations, 1 in six locations, and none in seven or more locations. The number of apoptosis proteins located in different number of locations can be displayed in Figure 1.

### 2.2. Feature Extraction

In order to develop a machine-learning-based predictor for protein subcellular localization prediction, one of the key steps is how to formulate a biological sequence with a discrete model or a feature vector that truly reflects the intrinsic relationship between proteins and their subcellular locations. However, it is not an easy job. Amino acid composition (AAC) is the simplest feature extraction method. Each protein is represented as a 20D feature vector, where the elements of the vector are the occurrence frequencies of the amino acids of the protein. However, AAC has an obvious shortcoming that only contains sequence features; therefore, the prediction performance might be considerably limited. To overcome this problem, Pseudo-amino-acid composition (PseAAC) is proposed based on amino acid composition, and it has almost penetrated into all the fields of protein attribute prediction, protein structural classes prediction [19, 20], super secondary structure prediction [21], protein subcellular locations prediction [22, 23], protein submitochondrial locations prediction [24], and so on. Meanwhile, the concept of PseAAC has also stimulated the generation of pseudofolding topological indices and pseudofolding lattice network [25, 26]. Inspired by PseAAC, pseudo-*k*-tuple-reduced-amino-acids composition (PseKRAAC) [27] is developed, which could simplify protein complexity, decrease the chance of overfitting, and improve prediction performance. In addition, the position-specific scoring matrix (PSSM) is adopted to describe the protein sequence evolution information according to the theory of species evolution. A protein with *L* sequence lengths can be represented as an *L*-row, 20-column matrix. The element (*i*,* j*) of PSSM represents the fact that the score of the amino acid in the *i*th position (*i* = 1, 2,…,* L*) of the sequence is changed to amino acid type* j* (*j *= 1, 2,…, 20) during the evolution process. The PSSM can be obtained by the PSI-BLAST to search the Swiss-Prot database. It also has been widely used in protein attribute prediction areas, including predicting protein subcellular localization [28], predicting protein-ATP binding residues [29], and predicting protein-protein interaction [30].

Gene Ontology (GO) database is established by the GO Consortium to provide a unified representation of genes and their products across all species. In GO database, the GO terms are used to describe the characteristics of genes and gene products, which contains three types: cellular component, molecular function, and biological process. The Gene Ontology Annotation (GOA) database annotates gene products and provides reference and evidence to support the annotations. The database can give a large and comprehensive research resource for proteomics. In GOA database, unique accession number (AC) of proteins in UniProtKB may correspond to different numbers of GO terms, which can be zero, one, or more GO terms, and one GO term may be related to zero, one, or many different ACs. It shows that the relationships between ACs and the GO terms may be many-to-many.

In recent years, several GO-based predictors have exhibited excellent performance in protein subcellular localization, such as iLoc-Plant [31], iLoc-Gpos [32], iLoc-Gneg [33], and Virus-ECC-mPLoc [34]. Furthermore, these predictors have fully proven that GO-based methods have superiority over sequence-based methods. However, there is some controversy or confusion about GO-based approaches for protein subcellular localization prediction: if a protein can find its cellular component GO terms, is it still needed to predict its subcellular location? Is that a solution to the prediction problem by creating a lookup table with the cellular component GO terms as the keys and the cellular components as the hashed values? Our previous work [32] and another research [35] have already illustrated the legitimacy of the GO-based predictors for protein subcellular localization. For readers' convenience, here we give a brief summary. For GO and non-GO predictors, their benchmark datasets were established based on the Swiss-Prot database, in which the subcellular locations of the proteins in the datasets were determined by experiments. The output of these GO-approach predictors was the subcellular location(s) by using the sequence information of the query protein alone as the input without needing any GO information. That is to say, there is no difference at all between the non-GO predictors and GO predictors in the requirement of the input. Additionally, the reason why the GO-based methods can perform excellent performance is that the features vectors in the GO space more accurately reflect the relationship between the proteins and their subcellular locations. Obtaining the locations of the query proteins by creating a lookup table using the cellular component GO terms and the cellular component categories has been demonstrated to be undesirable and leads to very poor prediction performance. In summary, we also applied the GO-based method in the current paper, and the details are given below.

Given a query protein *P*, it is entered to BLAST to search the Swiss-Prot database (released on 04 July 2016) for its homologous proteins. Collect these homologous proteins and put them into a set. The proteins in the set have some similar attributes such as structural conformations and biological functions as *P*. Select the accession numbers (AC) of homologous proteins as the keys to retrieve the relevant GO terms from the GOA database. Note that if the homologous proteins cannot be discovered or have any GO terms, then *P*'s own AC is to be used.

Using (1), protein *P* is represented as

(1) $$\begin{array}{c} P={\left[\;f_{1},f_{2},f_{3},\ldots ,f_{\mu },\ldots ,f_{\omega }\;\right]}^{T}, \end{array}$$

where T is a transpose operator; *ω* is the number of all GO terms in GO database. However, the number of GO terms has been increasing rapidly in recent years, and GO database (released on 23 July 2016) has included more than 20000 GO terms. If we use all the GO terms in GO database to formulate the feature vector, it will lead to the high-dimension disaster and time-costing problems. In the current paper, GO subspace was constructed to avoid these problems. For all apoptosis proteins in the dataset, we get their homologous proteins set and retrieve their GO terms as described above and put the GO terms into a set. After this process, all GO terms in the set form a GO Euclidean space with *ω* dimensions, where *ω* is equal to the number of GO terms in the set. GO subspace can be seen as a subset of all GO terms in GO database. For protein *P*, its GO feature vector is represented as (1) by mapping its GO terms to the GO subspace, where *f*_*μ*_ in the GO feature vector is defined as

(2) $$\begin{array}{c} f_{\mu }=\left\{\begin{array}{cc} g_{\mu } & \text{if GO hit}\\ 0 & otherwise, \end{array}\right. \end{array}$$

where *g*_*μ*_ is the number of occurrences of the *μ*th GO term, if the GO terms of protein *P* hits the *μ*th GO term. Note that, for each protein in the dataset, at least one AC has GO terms, where the AC may belong to protein itself or its homologies; therefore, naught vectors will not appear in the dataset. Naught vector is meaningless for prediction.

### 2.3. The Prediction Algorithm

Prediction of subcellular localization of multilocation apoptosis proteins can be regarded as a multilabel classification problem, where each subcellular location is represented as a class label. The binary relevance method (BR) is a frequently used strategy that converts the multilabel problem into several single-label classification problems. Given the multilabel training dataset *𝕊*, it contains *n* proteins classified into *c* subcellular locations. The dataset can be further grouped into *c* subsets according to the different locations: *𝕊* = *𝕊*_1_ ∪ *𝕊*_2_ ∪ ⋯ ∪  *𝕊*_*i*_ ∪ ⋯∪*𝕊*_*c*_, where *𝕊*_*i*_ is the subset containing the proteins belonging to the *i*th location. For the *i*th subcellular location, the training set can be represented as

(3) $$\begin{array}{c} T\left(\;i\;\right)=T^{+}\left(\;i\;\right)\cup T^{-}\left(\;i\;\right), \end{array}$$

where *𝕋*^+^(*i*) is the positive set of protein samples belonging to this location and *𝕋*^−^(*i*) is negative set that consists of the rest of the proteins; *𝕋*^+^(*i*) and *𝕋*^−^(*i*) are constructed as follows:

(4) T+i=Xp,+1 ∣ p∈Si,T−i=Xq,−1 ∣ q∉Si,

where **X**_*p*_ is the feature vector of protein *p* belonging to *𝕊*_*i*_ and **X**_*q*_ is the feature vector of protein *q* not belonging to *𝕊*_*i*_. BR method trains *c* independent binary classifier based on *𝕋*(*i*)  (*i* = 1, 2,…, *c*). Inputting a query protein, the prediction output is a *c*-dimensional score vector *y*, where *y*_*i*_ = +1 indicates that the protein belongs to the label *λ*_*i*_ or subcellular location *i* and *y*_*i*_ = −1 means that it does not belong to subcellular location *i*.

In this paper, a new multilabel prediction algorithm is proposed based on the binary relevance method (BR) strategy. Generally speaking, the proposed algorithm firstly selects the most discriminative features for the *c* subcellular locations, respectively and secondly constructs the classification models by using the *c* groups of label-specific features obtained via the above step. Specifically, we use Pearson's correlation coefficient (PCC) to select label-specific features for each subcellular location. PCC is a statistical method to measure the linear correlation between the two variables, whose value range is between −1 and +1. If the absolute value is close to 1, the linear correlation of the two variables is very high; otherwise, the value is close to 0; there is almost no linear correlation between them. PCC has been extensively used in biological data analysis [36]. Let *𝕏* and *𝕐* denote the feature vector space and the label score vector space, respectively, and they can be denoted as

**Figure 2:**
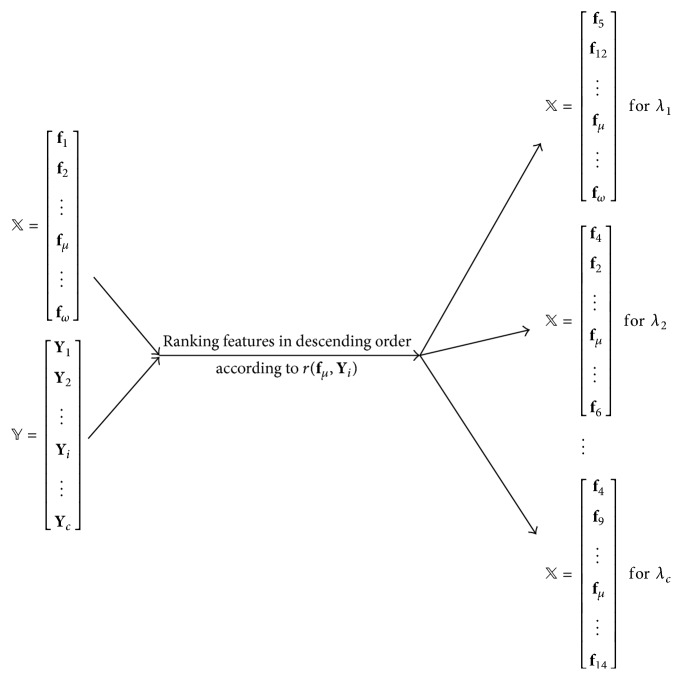
Schematic illustration of using Pearson's correlation coefficient (PCC) to rank features for each different class label.

(5) X=f1,f2,…,fμ,…,fω,Y=Y1,Y2,…,Yi,…,Yc,

where **f**_*μ*_ is the vector that consists of the *μ*th feature of all proteins and **Y**_*i*_ is the vector that is made up of label scores of all proteins for *λ*_*i*_; they are represented as:

(6) fμ=f1,μ,f2,μ,f3,μ,…,fk,μ,…,fn,μΤYi=y1,i,y2,i,y3,i,…,yk,i,…,yn,iΤ,

where *f*_*k*,*μ*_ is the *μ*th feature of the *k*th protein and *y*_*k*,*i*_ is the label score of the *k*th protein to *λ*_*i*_. The linear dependency between the *μ*th feature and class label *λ*_*i*_ is detected by

(7) $$\begin{array}{c} r\left(\;f_{\mu },Y_{i}\;\right)=\frac{\sum_{k=1}^{n}\left(f_{k,\mu }-\bar{f_{\mu }}\right)\left(y_{k,i}-\bar{Y_{i}}\right)}{\sqrt{\sum_{k=1}^{n}{\left(f_{k,\mu }-\bar{f_{\mu }}\right)}^{2}\sum_{k=1}^{n}{\left(y_{k,i}-\bar{Y_{i}}\right)}^{2}}}, \end{array}$$

where $\bar{f_{\mu }}$ and $\bar{Y_{i}}$ are mean values of **f**_*μ*_ and **Y**_*i*_, respectively. For each label, its label-specific features are constructed as follows: detect linear dependency between each feature and the current label, arrange the original features in descending order according to the linear dependencies, and then select first *K* features as label-specific features, where the value of *K* to each label may be different.  Figure 2 shows schematic illustration of using Pearson's correlation coefficient (PCC) to rank features for each different class label. In the process of classification models induction, BR strategy is used to induce binary classifier for each label. These binary classifiers are trained from the generated label-specific features other than the original features. For a query protein, similarly, its label-specific features instead of original features are used for prediction. In this paper, support vector machine (SVM) was used for training all the binary classifiers. SVM is a common binary classification algorithm and puts up some special advantages in the fields of nonlinear and high-dimensional pattern recognition.

**Figure 3:**
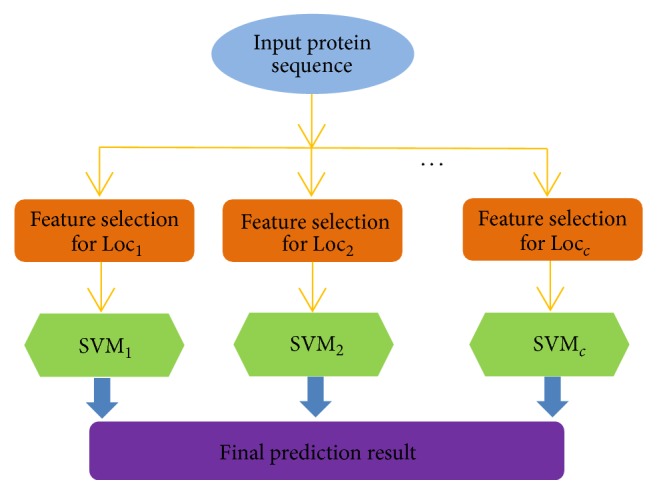
A flowchart to show how the MultiP-Apo predictor works. See the text for further explanation.

Finally, the entire predictor ever established via the above procedures is named MultiP-Apo, where “MultiP” stands for “multilocation prediction” and “Apo” stands for “apoptosis proteins.” To provide an intuitive picture, a flowchart is given in Figure 3 to illustrate the prediction process of MultiP-Apo.

### 2.4. Performance Measures

Predicting subcellular localization of multilocation apoptosis proteins belongs to the case of multilabel classification. It is well known that, for a multilabel classification system like the current system, performance metrics differ from those of traditional single-label classification system, because an example may have one or more class labels simultaneously. The performance metrics will be much more complicated for a multilabel classification system. To better reflect the multilabel capabilities of classifiers, these five measures, mlACC, mlPRE, mlREC, mlF1, and ACC, are used in this work, and they are defined as follows:

(8) mlACC=1m∑i=1mYi∩ZiYi∪Zi,mlPRE=1m∑i=1mYi∩ZiZi,mlREC=1m∑i=1mYi∩ZiYi,mlF1=2·mlREC·mlPREmlREC+mlPRE,ACC=1m∑i=1m1Yi≡Zi,

where *Y*_*i*_ is the set of true labels of each sample, *Z*_*i*_ is the set of predicted labels, *m* is the number of test samples, and |·| is the operator to count the number of the elements in the set. For the above five measures, the higher the measure values, the better the prediction performance. mlF1 is the harmonic mean of multilabel precision (mlPRE) and multilabel recall (mlREC), which takes the trade-off between mlPRE and mlREC into account to reflect the classification performance intuitively. ACC is a stringent measure that evaluates the overall correct rate of multilabel classification system. If true labels and predicted labels of an example are entirely identical, the value of 1(*Y*_*i*_ ≡ *Z*_*i*_) is 1; otherwise, it is 0. For a protein sample, only if all predicted locations are entirely identical to its true locations, it is considered to be correctly predicted. For example, a protein contains three subcellular locations; if the predicted result contains more than or less than three locations or the result has a location not belonging to the three true locations of the given protein, the prediction can be considered as incorrect. The readers can refer to the review article in [37] which has given a more detailed explanation about the meanings of these measures.

In statistical prediction, three common testing methods, independent dataset test, *k*-fold cross-validation, and jackknife cross-validation, are usually used for testing the generalization capabilities of predictors. Among them, the jackknife cross-validation is the most rigorous and bias-free testing method, as elucidated in a comprehensive review [38]. In the jackknife test, the dataset containing *N* proteins is divided into *N* subsets, where each subset is regarded as a test protein; the rest of *N* − 1 proteins are used as a training set. This procedure is repeated *N* times, and each time a different protein is selected as the test protein. The jackknife test has been increasingly and widely employed by researchers to examine the accuracy of various prediction methods [14–17, 21–24]. Hence, in the current paper, we also use the jackknife cross-validation to examine the prediction performance.

## 3. Results and Discussion

### 3.1. Evaluating Our Prediction Model on the Benchmark Dataset MSapo518

To demonstrate the efficiency of our proposed predictor, Table 2 compares the performance of our proposed predictor MultiP-Apo (using the label-specific features) with that of BrP-Apo (using original features) on the benchmark dataset MSapo518 by the jackknife test. Specifically, BrP-Apo used the BR strategy for training the prediction model, while our proposed predictor MultiP-Apo extended the BR strategy by utilizing label-specific features for prediction model. For a fair comparison, we used the same original features obtained in Section 2.2 and the same base classifier SVM for both MultiP-Apo and BrP-Apo. As can be seen from Table 2, MultiP-Apo performs impressively better than BrP-Apo in terms of mlACC, mlPRE, mlREC, mlF1, and ACC. Particularly, for the most objective and stringent criteria ACC, MultiP-Apo outperforms BrP-Apo by more than 15%. This is understandable because, in the basic BR strategy, for example, BrP-Apo, the same features are used to train each individual binary classifier for each subcellular location, leading to outputting many prediction errors. This problem can be overcome by using the label-specific features because it constructs the most discriminative features for each subcellular location, leading to a significant improvement on ACC. For the rest of the evaluation criteria, MultiP-Apo also significantly outperforms BrP-Apo, which is consistent with the aforementioned analysis demonstrating that taking the label-specific features into account can achieve higher prediction performance.

It should be noted that calculating and comparing the accuracy of each label is meaningless in a multilabel classification. Therefore, Table 3 listed the overall accuracies (ACCs) of apoptosis proteins with different number of labels (subcellular locations), and, for comparison, the ACCs by BrP-Apo are also shown in Table 3. As can be seen from Table 3, MultiP-Apo performs better than BrP-Apo significantly. In particular, for proteins with two subcellular locations, compared to BrP-Apo, the performance improvement of MultiP-Apo is close to 20%. We have noticed that the more subcellular locations the proteins have, the lower their ACCs are. Therefore, Table 3 can also show that using the label-specific features could enhance the prediction performance.

### 3.2. Effect of the Number of Homologous Proteins

**Figure 4:**
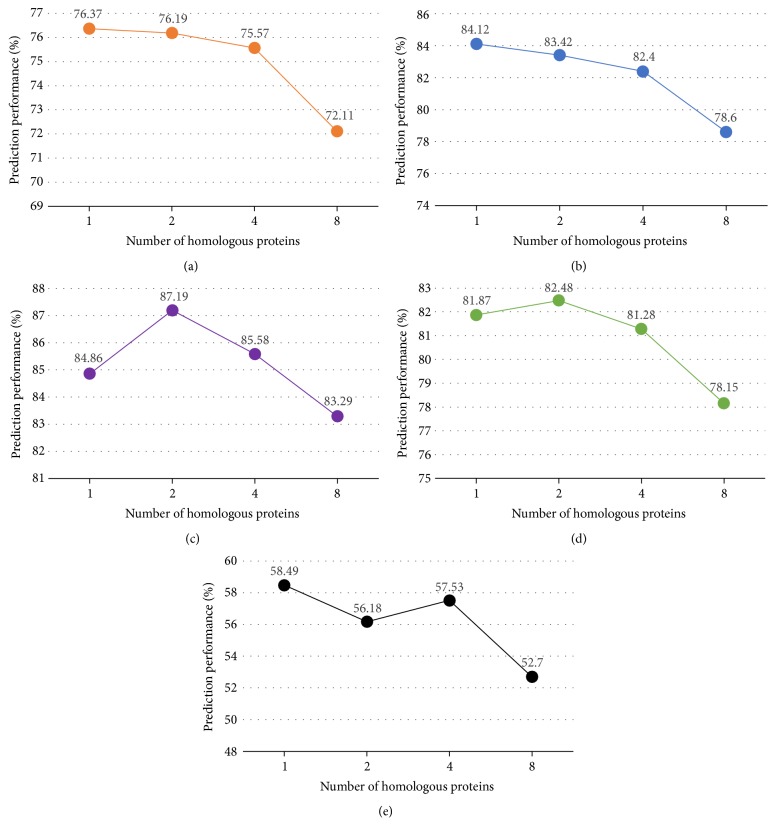
The graph shows how different numbers of homologous proteins affect the prediction performance (a) for the mlACC metric, (b) for mlPRE, (c) for mlREC, (d) for mlF1, and (e) for ACC.

In the section, we evaluate the performance of MultiP-Apo with different numbers of homologous proteins on the benchmark dataset MSapo518 by the jackknife test. The number of distinct GO terms can be different for different numbers of homologous proteins. Typically, the number of distinct GO terms increases with the number of homologous proteins. We select {1,2, 4,8} as the numbers of homologous proteins used here.  Figure 4 shows how the number of homologous proteins can affect the performance of MultiP-Apo. As can be seen from Figure 4, as the number of homologous proteins increases, the prediction performance of MultiP-Apo is generally decreased in terms of all performance metrics. Specifically, for absolute accuracy (ACC), the performance of using one homolog is remarkably better than that of using eight (58.49% versus 52.7%). This observation indicates that we should add the less number of homologous proteins because too many homologous proteins may bring in redundant and noisy information.

### 3.3. Comparison with the Existing Predictors for Apoptosis Proteins

As mentioned in Introduction, all the existing predictors can only be used to identify a single subcellular location of a query protein; none of them can deal with proteins with multiple subcellular locations. Nevertheless, it is still interesting to see if our proposed predictor could work better than the existing predictors based on the independent test using a new apoptosis protein dataset. The new apoptosis protein dataset was constructed by using the same criteria specified in Dataset. Moreover, to ensure that the proteins in the new dataset are really novel, the addition dates of these proteins should be later than the training proteins used in our proposed predictor and other existing predictors. Because the apoptosis protein datasets used for training MultiP-Apo and other predictors were created on 04 July 2016 and earlier, we selected the apoptosis proteins that were added to Swiss-Prot between 04 July 2016 and 15 May 2017. After that, 26 apoptosis proteins distributed in 8 subcellular locations were selected, of which 9 proteins are associated with one subcellular location, 9 with two locations, 6 with three locations, 1 with four locations, 1 with five locations, and none with six or more locations. In other words, 65% of the apoptosis proteins in the new dataset are located in multiple locations. The new dataset can also be downloaded from the MultiP-Apo server.

We compare our proposed predictor MultiP-Apo with the state-of-the-art predictor GO-DWKNN [15] on the new dataset by the independent test. Because GO-DWKNN is superior to the other existing predictors and only GO-DWKNN provides the online web server, we think the comparison would suffice. The prediction results of the two compared predictors are presented in Table 4. As can be seen from the table, MultiP-Apo performs significantly better than GO-DWKNN in terms of all performance metrics. Among the five metrics in (8), the ACC is the strictest and most harsh one; any overprediction or underprediction will lead to faulty results. The absolute accuracy (ACC) of our proposed predictor MultiP-Apo is more than 26% (absolute) higher than that of GO-DWKNN (46.15% versus 19.23%). This observation indicates that because MultiP-Apo is especially designed for dealing with apoptosis proteins with multiple subcellular locations, MultiP-Apo performs significantly better than GO-DWKNN in predicting subcellular locations of apoptosis proteins with both single and multiple sites.

## 4. Web Server

Since user-friendly and freely accessible web servers represent the future direction for developing practically more useful predictors, based on the above prediction method, we have developed an online web server for predicting multilabel apoptosis protein subcellular localization, called MultiP-Apo, at http://biomed.zzuli.edu.cn/bioinfo/multip-apo/. Even if there is no professional math and computer knowledge for the biologists, the prediction results can be also easily obtained for the query proteins.

## 5. Conclusion

Prediction of apoptosis protein subcellular localization is a challenging problem, and many outstanding predictors have been developed to solve this problem. However, there have been the following shortcomings in all the existing predictors: (1) for the proteins with multiple locations, they cannot completely predict all their subcellular locations; (2) so far no dataset contains the apoptosis proteins with multiple locations; (3) the machine learning algorithms used in these predictors are not suitable for dealing with the apoptosis proteins with multiple subcellular locations. In view of this, a multilabel predictor, namely, MultiP-Apo, is proposed in this paper, which is the first multilabel predictor for identifying subcellular locations of apoptosis proteins with single and multiple locations.

The main contributions of this paper can be summarized as follows: (1) we created the new benchmark dataset MSapo518 that contains 518 apoptosis proteins with both single and multiple subcellular locations and covers 8 subcellular locations; (2) we used the GO annotation information of the homology proteins of apoptosis proteins to formulate the feature vectors, and GO subspace was constructed to avoid the high-dimensional disaster by selecting a set of relevant GO terms from all the GO terms; (3) we proposed a novel multilabel algorithm by utilizing the label-specific features to perform multilocation prediction; (4) an online web server for MultiP-Apo is established which is freely accessible at http://biomed.zzuli.edu.cn/bioinfo/multip-apo/.

## Figures and Tables

**Table 1:** Breakdown of the apoptosis protein benchmark dataset MSapo518.

Order	Compartment	Number of proteins
1	Cytoplasm	244
2	Membrane	126
3	Secreted	36
4	Mitochondrion	107
5	Nucleus	207
6	Endosome	12
7	Endoplasmic reticulum	47
8	Golgi apparatus	25

**Table 2:** Performance comparison of MultiP-Apo with BrP-Apo on the benchmark dataset MSapo518 by the jackknife test.

Measure	MultiP-Apo (%)	BrP-Apo (%)
mlACC	76.37	62.84
mlPRE	84.12	71.10
mlREC	84.86	74.56
mlF1	81.87	69.61
ACC	58.49	42.08

**Table 3:** A comparison of the overall accuracies (ACCs) by MultiP-Apo and BrP-Apo for proteins with different number of subcellular locations.

Number of locations	Number of proteins	The overall accuracy (ACC)	
		MultiP-Apo (%)	BrP-Apo (%)
1	303	68.65	50.83
2	155	56.13	36.77
3	52	15.38	13.46
4	6	0	0
5	1	0	0
6	1	0	0

**Table 4:** Multilabel performance comparison of MultiP-Apo with GO-DWKNN on a new dataset by the independent test.

Measure	MultiP-Apo (%)	GO-DWKNN (%)
mlACC	69.17	48.53
mlPRE	90.38	88.46
mlREC	72.05	48.53
mlF1	77.07	59.87
ACC	46.15	19.23
